# Integrated Evaluation of the Potential Health Benefits of Einkorn-Based Breads

**DOI:** 10.3390/nu9111232

**Published:** 2017-11-11

**Authors:** Fabiana Antognoni, Roberto Mandrioli, Alessandra Bordoni, Mattia Di Nunzio, Blanca Viadel, Elisa Gallego, María Paz Villalba, Lidia Tomás-Cobos, Danielle Laure Taneyo Saa, Andrea Gianotti

**Affiliations:** 1 Department for Life Quality Studies, University of Bologna, Corso Augusto 237, 47921 Rimini, Italy; fabiana.antognoni@unibo.it (F.A.); roberto.mandrioli@unibo.it (R.M.); 2 Department of Agri-Food Sciences and Technologies, University of Bologna, Piazza Goidanich 60, 47521 Cesena, Italy; alessandra.bordoni@unibo.it (A.B.); danielle.taneyosaa2@unibo.it (D.L.T.S.); andrea.gianotti@unibo.it (A.G.); 3 Department of New Products and Department of Bioassays, AINIA Technological Centre, c/Benjamín Franklin 5-11, Paterna, 46980 Valencia, Spain; bviadel@ainia.es (B.V.); egallego@ainia.es (E.G.); mpvillalba@ainia.es (M.P.V.); ltomas@ainia.es (L.T.-C.)

**Keywords:** einkorn, wheat, carotenoids, bread, phenolic acids, sourdough fermentation, in vitro digestion, Caco-2 cells, anti-inflammatory effect

## Abstract

Nowadays the high nutritional value of whole grains is recognized, and there is an increasing interest in the ancient varieties for producing wholegrain food products with enhanced nutritional characteristics. Among ancient crops, einkorn could represent a valid alternative. In this work, einkorn flours were analyzed for their content in carotenoids and in free and bound phenolic acids, and compared to wheat flours. The most promising flours were used to produce conventional and sourdough fermented breads. Breads were in vitro digested, and characterized before and after digestion. The four breads having the best characteristics were selected, and the product of their digestion was used to evaluate their anti-inflammatory effect using Caco-2 cells. Our results confirm the higher carotenoid levels in einkorn than in modern wheats, and the effectiveness of sourdough fermentation in maintaining these levels, despite the longer exposure to atmospheric oxygen. Moreover, in cultured cells einkorn bread evidenced an anti-inflammatory effect, although masked by the effect of digestive fluid. This study represents the first integrated evaluation of the potential health benefit of einkorn-based bakery products compared to wheat-based ones, and contributes to our knowledge of ancient grains.

## 1. Introduction

Several studies have shown a clear correlation between the consumption of wholegrain and a reduced risk of cardiovascular diseases [1,2], diabetes [3], and some types of cancer [4]. The beneficial properties of wholegrain are mainly ascribed to their micronutrient and phytochemical content [5,6,7]. Cereals are among the richest food in phenolic acids, their content being comparable with or even higher than that found in berries, fruits, and vegetables [8]. In addition, some cereals are rich in lutein and zeaxanthin [9,10]. Micronutrients and phytochemicals are chiefly concentrated in the outer layers of grains [11], and this could explain the preventive effects associated with high wholegrain consumption [12].

Nowadays, the higher nutritional value of wholegrain compared to refined ones is recognized [13], and there is an increasing interest in ancient crops as source of wholegrain flours [14].

Einkorn (*Triticum monococcum* L. ssp. *monococcum*) is an ancient crop. Compared to polyploid wheats it has a higher content of proteins, polyunsaturated fatty acids, fructans, and phytochemicals as tocols, carotenoids, alkylresorcinols, phytosterols, and a lower α-, β-amylase and lipoxygenase activities [15]. In addition, einkorn expresses very few T-cell stimulatory gluten peptides [16]. Einkorn could represent a valid alternative for producing functional baked products.

In bakery, processing could contribute to functionality [17,18]. Sourdough fermentation, involving the inter-relation between microbial metabolism and cereal enzymes, has been shown to greatly affect the functional features of leavened baked goods [19]. This type of fermentation may produce new nutritionally active molecules such as functional peptides and amino acid derivatives [20,21], deriving from either the bacterial hydrolytic activity [20] or from their own synthetic pathways [22]. To exert a positive action in the human body, bioactive compounds must be hydrolyzed from the food matrix, and be absorbed in the intestine. The bioaccessibility of bioactive compounds, i.e. the percentage released from the food matrix and made available for uptake by the intestinal mucosa, is an important parameter that can be influenced by many different factors including the food matrix and the food processing [23,24]. Fermentation by lactic acid bacteria may improve nutrient bioaccessibility and produce compounds with anti-oxidant and anti-inflammatory activity [19]. Sourdough lactic acid bacteria have been reported to release or synthesize antioxidant and anti-inflammatory peptides during fermentation of cereal flours [20].

In this work, different wheat and einkorn flours were analyzed for their content in carotenoids and phenolic acids. The richest in these functional compounds were selected, and used to bake breads with two different fermentation procedures (conventional and sourdough).

Breads were digested in vitro using a dynamic gastro-intestinal digestor, and characterized before and after digestion. Based on integrated results, four breads were selected, and the product of their intestinal digestion was supplemented to Caco-2 intestinal cells. Cells were exposed to inflammatory stress, and the effect of supplementation on different inflammation markers was assessed.

Overall, this study has evaluated how the type of flour and the type of fermentation can influence the nutritional features of bread, and the bioaccessibility and anti-inflammatory effects of its functional compounds. The combination of different results provides an integrated vision supporting the possible health benefits of einkorn-based bread.

## 2. Materials and Methods

### 2.1. Materials

Phenolic acids (4-hydroxybenzoic acid, caffeic acid, chlorogenic acid, ferulic acid, gallic acid, p-coumaric acid, synapic acid, syringic acid, and trans-cinnamic acid) pure standards (≥99.5% purity) in powder form; HPLC-grade methanol, acetonitrile, acetone, diethyl ether, ethyl acetate and water; phosphoric acid (85–87%, *w*/*w*), hydrochloric acid (37%, *w*/*w*) monobasic sodium phosphate (≥98%), sodium hydroxide beads (≥98%) were produced by Sigma-Aldrich Italia (Milan, Italy). Stock solutions (1 mg/mL) of phenolic acids were prepared by dissolving 10 mg of each pure substance in 10 mL of methanol. Standard solutions were obtained by diluting stock solutions with the mobile phase (initial composition). Certified stock solutions of carotenoids in acetonitrile (520 µg/mL neoxanthin; 752 µg/mL violaxanthin; 590 µg/mL antheraxanthin; 830 µg/mL zeaxanthin; 771 µg/mL lutein; 1033 µg/mL β-cryptoxanthin; 894 µg/mL β-carotene) were purchased from DHI (Hørsholm, Denmark) and diluted with methanol before injection. When stored at −20 °C in the dark, stock solutions were stable for at least one month (as assessed by HPLC); standard solutions were prepared fresh every day.

An XS Instruments (Carpi, Italy) pH 50 pHmeter, a Thermo Scientific CL10 centrifuge, an IKA (Staufen, Germany) A11 Basic knife mill and a M. Christ Alpha 1–4 LD freeze dryer were used.

Caco-2 and HEK-Blue™ IL-6 cells were obtained from Sigma-Aldrich (Saint Louis, MO, USA) and InivoGen (San Diego, CA, USA), respectively. Earle’s Balanced Salt Solution (EBSS), Dulbecco’s Modified Eagle’s Medium (DMEM), Fetal Bovine Serum (FBS), penicillin, streptomycin, L-glutamine were from GE Healthcare (Little Chalfont, UK). Non-Essential Amino Acids (NEAA), sodium pyruvate and Fungizone were purchased from Thermo Fisher Scientific (Waltham, MA, USA).

### 2.2. Flour Samples

Refined wheat flours (*Triticum aestivum* L.; standard flours, SF), including two Spanish types (550 S and 650 S) and two Polish types (550 P and 650 P), were provided by INDESPAN (Valencia, Spain) and VINI (Rogoznik, Poland), respectively. Einkorn flours (or ancient flour, AF) including organic whole and refined flours obtained from *Triticum monococcum* L. var. Monlis, were provided by Prometeo (Urbino, Italy).

### 2.3. Lactic Acid Bacteria (LAB) Strains and Sourdough Starter Preparation

*Lactobacillus plantarum* 98a, *Lactobacillus sanfranciscensis* BB12, *Lactobacillus brevis* 3BHI strains, belonging to the Department of Agricultural and Food Science and Technology (DISTAL) of the University of Bologna, were used. LAB strains were grown separately in Man Rogosa Sharpe (MRS) broth (Oxoid, Milan, Italy) at 37 °C for 24 h. Cells were harvested by centrifugation at 4000× *g* for 10 min, and washed twice with sterile water. To prepare sourdough, 600 g of flour were gently mixed with 270 mL of water and inoculated with 80 mL of a water suspension of each separately grown strain. The inoculated dough was incubated at 30 °C for 24 h to obtain a mature sourdough starter.

### 2.4. Fermentation and Baking Processes

Two types of fermentation processes were adopted: (i) conventional fermentation (CF) based on a commercial compressed yeast (baker’s yeast), (ii) fermentation with sourdough obtained from wheat flour (SSF) or einkorn flour (SAF). Conventional fermentation was performed by baker’s yeast (2.5% flour basis) at 30 °C for 1.5 h. Sourdough fermentation was obtained by the addition of the sourdough starter described above (around 30% of total dough). The inoculum level in the final dough was approximately 4 × 10^8^ CFU/g for each LAB strain. The final dough was finally added by baker’s yeast and fermented as described for conventional fermentation. Water was added to obtain DY = 250 and salt 2 g/100 g of flour. The fermented doughs were baked at 195 °C for 45 min in an industrial oven by VINI Company (Rogoznik, Poland).

Six experimental breads were thus obtained (Table 1).

### 2.5. In Vitro Digestion of Bread Samples

The six experimental breads were digested in vitro using a Dynamic Gastrointestinal Digestor (DGD). DGD is a multicompartment, computer-controlled system that simulates the biological environment in the human stomach and the small intestine [25]. The digestion process was performed on 50 g of experimental bread or 50 g of water (blank digestion) for 360 min (120 min in the stomach and 240 min in the small intestine) at 37 °C. It included several consecutive enzymatic treatments: saliva secretions (α-amylase), gastric secretions (pepsin and lipase) at acid pH, and intestinal secretions (bile and pancreatin) at neutral pH. The secretion of digestive juices, the pH adjustment and the gradual emptying of the stomach and small intestine, were simulated according to physiological data [26,27]. To minimize photooxidation, all experiments were performed with continuously flushing nitrogen gas and protected from light. The intestinal digesta obtained in each digestion were centrifuged at 5000× *g* at 4 °C for 45 min, and the supernatants filtered through 0.22 µm pore cellulose filters to have the aqueous fractions containing bioaccessible analytes [28]. Digesta were frozen and stored at −20 °C until their analysis.

### 2.6. HPLC Determination of Phenolic Acid Content

Bread samples were freeze-dried and finely ground in a knife mill for 4 × 30 s periods. Flour samples were directly used for extraction, while in vitro digesta were freeze-dried before extraction. Phenolic acids were extracted from samples according to the protocol by Moore et al. [29], with modifications. A 2-g aliquot of powdered sample was transferred in a knife mill with 20 mL of a methanol/acetone/water (7/7/6, *v*/*v*/*v*) mixture; after a 30 s mixing, the mixture was centrifuged for 5 min at 1400× *g*. Three fractions were extracted: two (free, soluble conjugated) from the sample supernatant and one (insoluble bound) from the pellet. Digested samples presented no pellet, thus only two fractions (free and soluble conjugated) were extracted from them.

#### 2.6.1. Free Phenolic Acids (FREE Fraction)

One mL of the supernatant was mixed with 10 mL of 0.01 N HCl and extracted thrice with an equal volume of a diethyl ether/ethyl acetate (1/1, *v*/*v*) mixture. The organic extracts were merged, brought to dryness in a rotary evaporator, and re-dissolved with 2 mL of methanol.

#### 2.6.2. Soluble-Conjugated Phenolic Acids (SC Fraction)

Eight mL of the supernatant were mixed with 2 mL of 10 M NaOH and hydrolyzed under nitrogen flow and constant stirring for 1 h. The solution was then acidified to pH 2 with 12 M HCl and subjected to the extraction procedure already described for the FREE fraction.

#### 2.6.3. Insoluble-Bound Phenolic Acids (IB Fraction)

A 0.5 g aliquot of the pellet was mixed with 40 mL of 2 M NaOH and hydrolyzed under nitrogen flow and constant stirring for 1 h. The sample was then centrifuged for 20 min at 1400× *g*; the supernatant was acidified to pH 2 with 12 M HCl and then subjected to the extraction procedure already described for the FREE fraction.

#### 2.6.4. HPLC Analysis

20 µL of each extract were injected into the HPLC system (Jasco, Tokyo, Japan; PU-4180 pump, MD-4015 PDA detector, AS-4050 autosampler). The stationary phase was an Agilent (Santa Clara, CA, USA) Zorbax Eclipse Plus C18 reversed-phase column (100 mm × 3 mm I.D., 3.5 μm).

The chromatographic method for the analysis of phenolic acids was adapted from Mattila et al. [30]. Gradient elution was carried out with a mixture of acidic phosphate buffer and acetonitrile flowing at 0.7 mL/min. The signals at 254, 280 and 329 nm were used for analyte quantitation. The recovery values of phenolic acids in spiked samples ranged from 78.8 to 92.2% (RSD < 9.8%, *n* = 6).

### 2.7. HPLC Determination of Carotenoid Content

Flour samples were directly used for extraction, while bread samples were freeze-dried and finely ground in a knife mill for 4 × 30 s periods. As well, the products of in vitro bread digestion were freeze-dried before extraction. Carotenoids were extracted from samples according to the following protocol: 5 g of flour or freeze-dried bread samples were mixed for 30 s in a knife mill with 20 mL of an ethanol/water (80/20, *v*/*v*) mixture, left to rest for 20 min, then mixed again for 30 s and centrifuged for 10 min at 4000× *g*. Freeze-dried digested samples (0.5 g) were extracted with 2 mL of the ethanol/water mixture. The supernatant was filtered and 20 µL of this solution were injected into the HPLC system (see Section 2.6). The method for carotenoid analysis was adapted from Hidalgo et al. [31]. Composition and flow rate gradient elution was carried out with a mixture of water and methanol/tetrahydrofuran (50/50, *v*/*v*). The signal at 450 nm was used for quantitative purposes. The recovery values of carotenoids in spiked samples ranged from 80.1 to 90.3% (RSD < 11.1%, *n* = 6).

### 2.8. Oxygen Radical Absorbance Capacity (ORAC) Assay

The assay was carried out on bread sample extracts using a Perkin Elmer (Turku, Finland) Viktor X3 multilabel plate reader, essentially as described by Moore et al. [29], with major modifications. For the setup of calibration curves, 210 µL of 10 nM fluorescein and 35 µL of Trolox at different concentrations (10–500 µM), or 35 µL of 10 mM phosphate buffer (blank solution), were introduced into each well. For sample analysis, the Trolox solution was replaced by 35 µL of suitably diluted sample. The plate was incubated at 37 °C for 5 min, then 35 µL of 240 mM 2,2′-Azobis(2-methylpropionamidine) dihydrochloride (AAPH) were added. The fluorescence emission intensity of each well was monitored for 150 min at 550 nm, exciting at 495 nm, while thermostating at 28 °C. Trolox equivalents (TE) were calculated from the relative area under the curve of the emission intensity vs. time plot.

### 2.9. Caco-2 Cell Culture and Supplementation

Caco-2 cells were kept at 37 °C, 5% CO_2_ in EBSS supplemented with 20% FBS, 100 U/mL penicillin, 100 mg/mL streptomycin, 2 mM L-glutamine, 1% NEAA, 1mM sodium pyruvate, and 0.4% Fungizone. Medium was changed every two days. After about 10 days, at 80% of confluence, cells were split (1:2–1:4) on a new flask (75 cm^2^). To perform the different assays, cells were seeded in 24-multiwell plates at 80,000 cell/well in DMEM without phenol red, 10% FBS, 100 U/mL penicillin, 100 mg/mL streptomycin, 2 mM L-glutamine, 1% NEAA. After 7 days, in preliminary experiments some cells were supplemented with the product of the blank digestion (1:25 *v*/*v*) for 24 h (B) while other cells received no supplementation (controls-C). In further experiments, after 6 h of supplementation with the digested samples (1:25 *v*/*v*), the product of the blank digestion (1:25 *v*/*v*) or no supplementation, inflammation was induced by exposure to IL-1β (10 ng/mL) for 18 h.

### 2.10. Cytokine Quantification in Caco-2 Cells

The level of the pro-inflammatory IL-6 and IL-8 was estimated in cell media by AlphaLISA kits (IL-6 and IL-8 Immunoassay Research Kits; Perkin Elmer Inc., Waltham, MA, USA) following the manufacturer’s instructions. 96 microwell plates (96 1/2 AreaPlate from Perkin Elmer) were used and read using an EnSpire™ plate reader from Perkin Elmer. Data were interpolated in a standard curve of IL-6 and IL-8 and results expressed as the percentage of the value obtained in control, not stimulated cells (assigned as 100%).

IL-6 secretion by Caco-2 cells was also indirectly evaluated using HEK-Blue™ IL-6 cells (Invivogen, San Diego, CA, USA). These cells were generated by stable transfection of HEK293 cells with the human IL-6R gene and a STAT3-inducible SEAP reporter gene. Upon IL-6 stimulation, HEK-Blue™ IL-6 cells trigger the activation of STAT3 and the subsequent secretion of SEAP, which can be monitored using the colorimetric reactive QUANTI-Blue™ (Invivogen, San Diego, CA, USA). Briefly, HEK cells were grown in DMEM, 10% (*v*/*v*) FBS, 50 U/mL penicillin, 500 µg/mL streptomycin, 100 µg/mL normocin, 2 mM L-glutamine, and seeded in 96 multiwell plates at 50,000 cell/well. Cells were incubated at 37 °C and 5% CO_2_ for 24 h with direct exposure to the media of Caco-2 cells grown in the different experimental conditions. After incubation, activation of JAK-STAT pathway was evaluated by QUANTI-Blue™ following manufacturer’s instructions.

### 2.11. Statistical Analysis

Statistical analysis was performed using the one-way Analysis of Variance (ANOVA) with Tukey’s Multiple Comparison Tests. The two-way ANOVA was used to evaluate the influence of two factors (flour type, fermentation) on functional compound levels. Ferulic acid content and ORAC activity were correlated using Pearson’s correlation test.

## 3. Results and Discussion

### 3.1. Carotenoid and Phenolic Acid Profile in Einkorn and Wheat Flours

**Figure 1:**
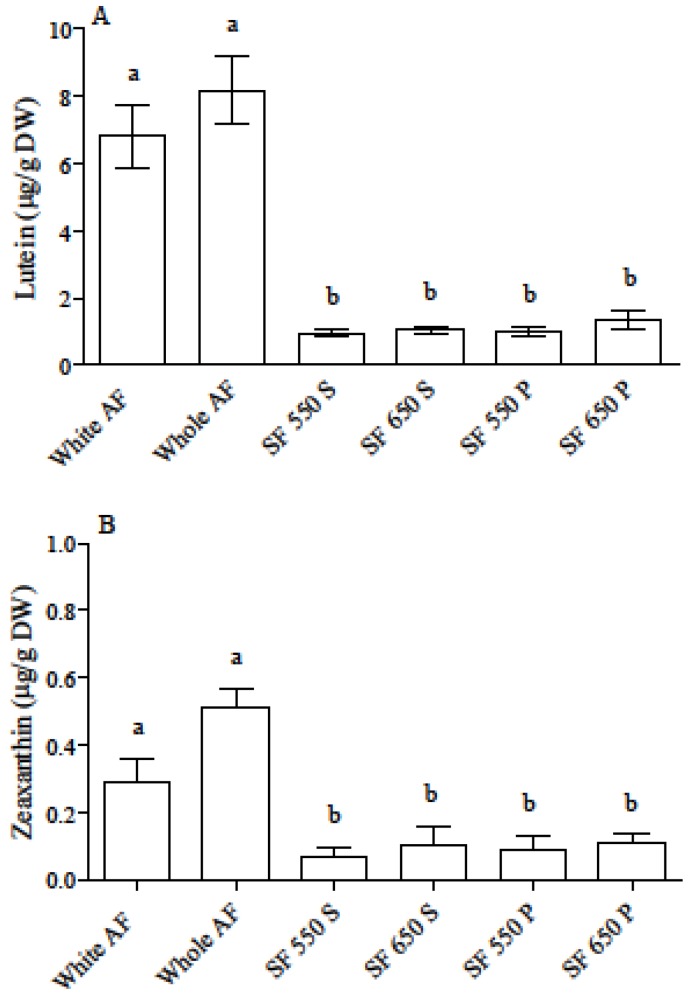
Lutein (**A**) and zeaxanthin (**B**) content in AF and SF samples. Results are the mean ± SD of two independent experiments with duplicate samples. Different letters indicate significant differences at *p* < 0.05. AF = ancient flour; SF = standard flour; S = Spanish flour; P = Polish flour.

Lutein and zeaxanthin were the only carotenoids detected in all flour samples, the former being by far more abundant than the latter. AF flour carotenoid levels were within expected ranges [32] and 7-fold higher than those of SF flours, as previously reported [33]. Within AF flours, the white type had a slightly, but not significantly, lower lutein and zeaxanthin content than the whole type. This confirms the high contribution of endosperm to carotenoid levels in the seed [34]. No significant differences in carotenoid contents were found between Spanish and Polish wheat flours (Figure 1).

About phenolic acids, ferulic acid was by far the most abundant one, followed by p-coumaric acid (Table 2). Other phenolic acids were present at lower levels, as already reported [6,35]. As expected, ferulic acid, which is linked to the cell wall components, occurred mostly in the IB form. AFs showed an about 4-fold higher ferulic acid content in the bound form compared to white SFs; between AFs, the whole type had the highest ferulic acid content. The difference between the whole and the white AF flours was not as evident as expected. Indeed, 22% less ferulic acid was present in the latter compared to the former. This can be explained by the fact that AF white flour was stone-ground, a process that maintained a rather high fiber content.

About white SF, Spanish and Polish samples showed similar ferulic acid contents. The levels of ferulic acid determined in our white SFs were in accordance to those reported by Mattila et al. [30], even though other authors found lower levels [11]. These discrepancies are probably due to the different conditions under which the cereals were grown and to different methodological procedures for phenolic acid analysis. p-Coumaric acid was found at much lower levels than ferulic acid, and was more uniformly distributed among the three fractions. White SF showed a ca. 5 fold higher free p-coumaric acid content than AF (Table 2).

### 3.2. Carotenoid and Phenolic Acid Profile in AF and SF Breads Prepared by Different Fermentation Processes

Based on results from the carotenoid and phenolic acid analyses, within each type (AF and SF) the two flours with the highest content in carotenoids and ferulic acid (whole AF and SF 650 P) were chosen for the preparation of breads using either conventional or sourdough fermentation.

**Figure 2:**
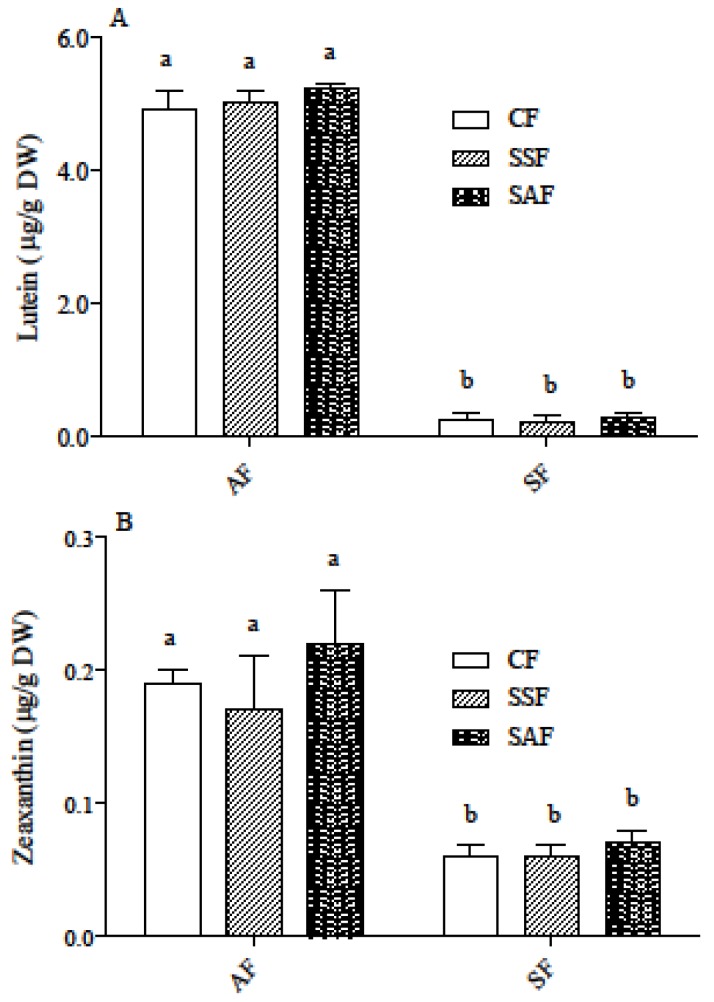
Lutein (**A**) and zeaxanthin (**B**) content in AF and SF breads prepared by CF or SSF or SAF as the starter flour. Results are the mean ± SD of two independent experiments with duplicate samples. Different letters indicate significant differences at *p* < 0.05. AF = ancient flour; SF = standard flour; CF = conventional fermentation; SSF = sourdough obtained from standard flour; SAF = sourdough obtained from ancient flour.

Carotenoid content in the different breads is shown in Figure 2. Lutein and zeaxanthin were the major carotenoids detected, zeaxanthin levels being much lower than lutein levels. Degradation of carotenoids during breadmaking was different in AF breads than in SF ones. In the former ones, lutein and zeaxanthin levels ranged from 58% to 76% of the levels in flour, while in the latter ones a lower percentage of carotenoids remained (16–20%).

Leenhardt et al. [36] investigated changes in carotenoid content during whole wheat and einkorn breadmaking and demonstrated that the kneading stage, besides the baking process itself, caused major carotenoid losses. This is due to the conspicuous incorporation of oxygen in the dough occurring in this phase, which facilitates the lipoxygenase (LOX)-catalyzed oxidation of polyunsaturated fatty acids, with co-oxidation of carotenoids. As previously reported [36], fermentation seems to have very little effect on carotenoid content, possibly due to oxygen consumption by baker’s yeast, which prevents LOX-mediated carotenoid degradation. Einkorn grains usually have a much lower LOX activity than wheat grains [37], and this could explain the lower degradation observed in AF breads.

Sourdough fermentation did not significantly affect carotenoid content compared to the conventional procedure (Figure 2). Thus, two-way ANOVA indicates that changes in carotenoid levels among bread samples were only due to the flour type (*p* < 0.001), and neither the fermentation procedure nor the interaction between flour and fermentation represented significant sources of variance (data not shown).

Several changes in functional compounds and nutrients occur in baked goods fermented by LAB, but little information is available concerning carotenoids. Considering the much longer duration of sourdough fermentation, which causes higher oxygen incorporation, a more pronounced degradation of carotenoids would be expected in LAB-fermented breads. In this study, LAB used to ferment experimental breads were selected based on their suitability to increase the quality of bakery products [38]. Lactobacillus *plantarum* 98a has been reported to increase the delivery of antioxidant compounds [39]. It is possible that the acidic environment and/or the microbial metabolism somehow prevented the activity of carotenoid-degrading enzymes. In addition, the production of new bioactive compounds in sourdough-fermented goods may derive from specific bacterial synthetic pathways. For example, *Lactobacillus plantarum* strains have been shown to exhibit a deep yellow pigmentation when cultured as isolated colonies [40] and this species can synthesize the yellow C30 carotenoid 4,4-diaponeurosporene [41].

**Figure 3:**
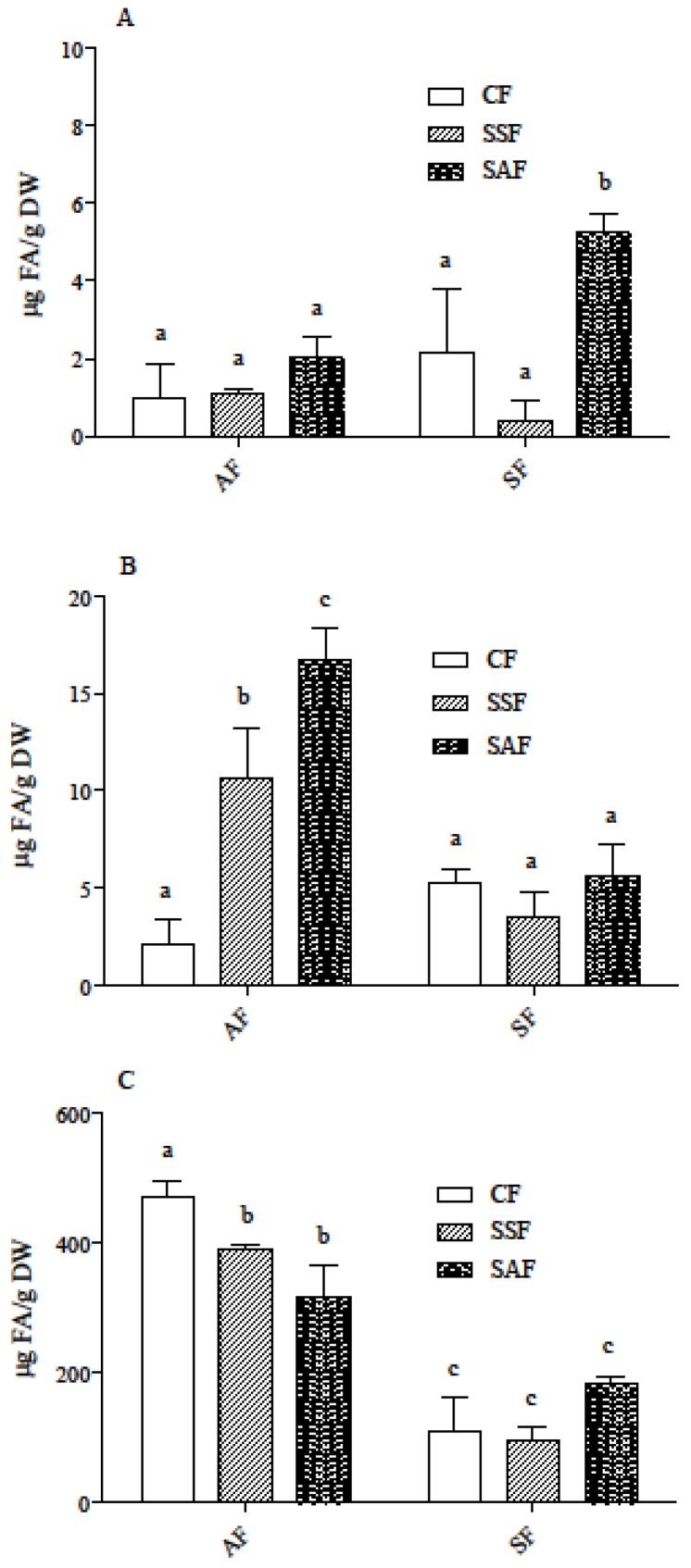
Ferulic acid content in the FREE (**A**), SC (**B**), and IB (**C**) fractions obtained from AF and SF breads prepared by CF or SSF or SAF as the starter flour. Results are the mean ± SD of two independent experiments with duplicate samples. Different letters indicate significant differences at *p* < 0.05. AF = ancient flour; SF = standard flour; CF = conventional fermentation; SAF = sourdough obtained from ancient flour; SSF = sourdough obtained from standard flour.

In all breads, ferulic acid was the most abundant phenolic acid and the major contribution came again from the IB fraction (>90% of the total) (Figure 3). The effect of sourdough fermentation was different depending on the type of flour used: in AF breads, LAB fermentation largely maintained the original levels of ferulic acid (11 and 17 µg/g in breads vs. 18 µg/g in flour), while causing a significant decrease in the bound fraction. Conversely, in SF breads no significant changes were found, either in the SC or in the IB form of ferulic acid, while a significant increase was observed in free ferulic acid when bread was prepared by sourdough fermentation with AF as the starter flour.

Thus, two-way ANOVA showed that both factors, flour type and fermentation, as well as their interaction, were significant in determining the variance in ferulic acid content among the different breads (*p* < 0.05 for the FREE fraction, and *p* < 0.01 for the other fractions). Fermentation gave the major contribution to the variance of total soluble ferulic acid content (Free + SC, *p* < 0.001). Total ferulic acid levels were similar in flours and in the corresponding baked products, thus suggesting that the breadmaking process did not alter phenolic acids to a significant extent. Free phenolic compounds and free ferulic acid were reported to increase in rye bran fermentations started with baker’s yeast [42] and in LAB-fermented wholemeal rye, oats and barley [43,44]. This has been related to the use of starter cultures endowed with feruloyl esterase activity. However, the effect of cultures on phenolic acid content, or lack thereof, was reported to be highly strain-specific for different reasons [44,45]. Intrinsic feruloyl esterase activity in cereals also contributes to phenolics metabolism [19], and a different effect in barley and oat groat flours fermented by the same strain was reported [44]. Thus, the observed decrease in IB ferulic acid in AF breads accompanied by a significant increase in the SC fraction may be explained by the binding of free ferulic acid produced by the hydrolysis of the bound form with low molecular weight molecules (such as small peptides and amino acids) produced by the fermentation process [46]. The different pattern of conversion of phenolic acids in AF and SF breads can be due to a different contribution of endogenous cereal feruloyl esterase activity from the bran component of AF activated during fermentation.

**Figure 4:**
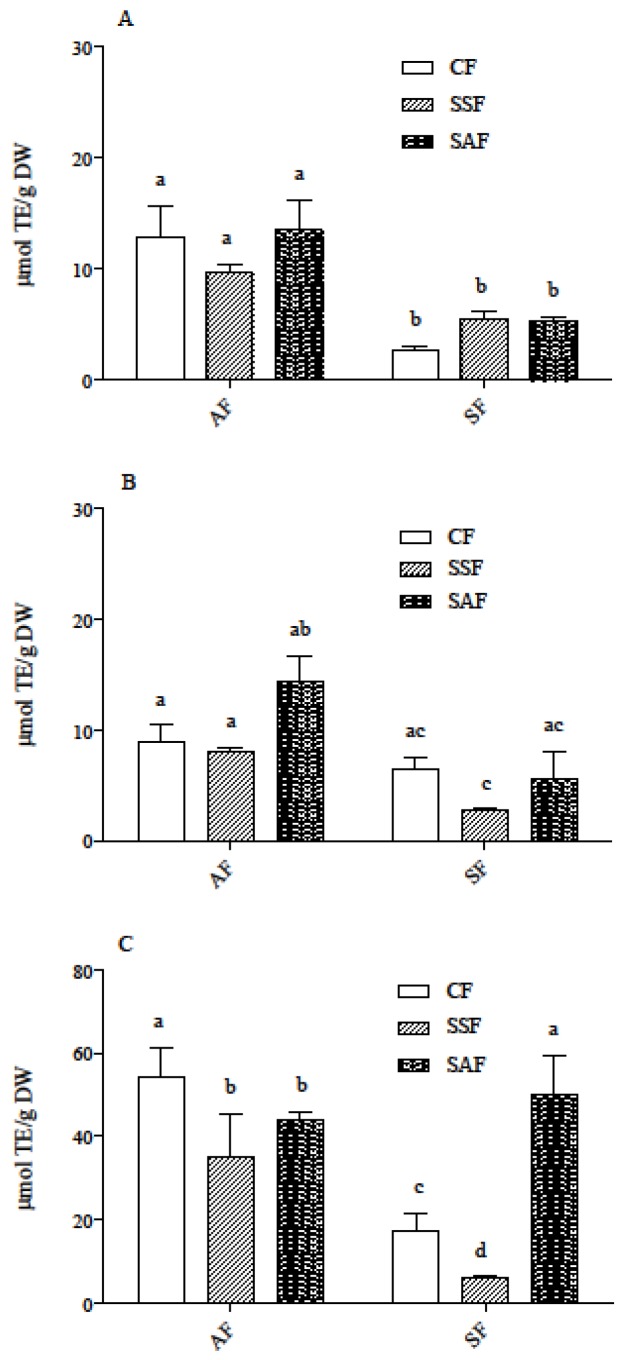
ORAC of extracts containing FREE (**A**), SC (**B**), and IB (**C**) phenolic acid fractions of bread samples. AF and SF breads were prepared by CF or SSF or SAF as the starter flour. Results are expressed as µmol TE/g DW and are the mean ± SD of two independent experiments with duplicate samples. Different letters indicate significant differences at *p* < 0.05. AF = ancient flour; SF = standard flour; CF = conventional fermentation; SAF = sourdough obtained from ancient flour; SSF = sourdough obtained from standard flour.

ORAC assays show that IB fraction had the highest activity, while FREE and SC had similar and lower activity (Figure 4). Nevertheless, the differences among IB, SC, and FREE fractions in ORAC activity were much lower than those in ferulic acid content, which was present, in the IB fraction, at levels up to 200 times higher compared to SC (Figure 3 and Figure 4). Correlation between ORAC and ferulic acid content in the SC and IB fractions was evaluated using Pearson’s correlation test, and only a moderate correlation (Pearson r values = 0.71 and 0.60, respectively) was found. This is not surprising and may be explained by the fact that extracts from these fractions are most probably complex mixtures, containing different classes of molecules, interacting among each other through synergism and/or antagonism, which can yield unpredictable antioxidant activity [47]. Moreover, the different classes of compounds in the extracts could have a reaction mechanism different from the hydrogen atom transfer, which the ORAC assay is based on. Both flour type and fermentation procedure represented a significant source of variation for radical-scavenging activity in SC and IB fractions, while the interaction between the two factors was not significant (data not shown).

### 3.3. Carotenoids and Phenolic Acids in In Vitro Digested Samples

Carotenoids and phenolic acids in the soluble fraction obtained after in vitro digestion of the bread samples represent the bioaccessible compounds, i.e., the amounts of compounds released by the digestion process and made available for absorption. As can be seen in Table 3, the percentage of bioaccessible lutein and zeaxanthin was rather high, reaching more than 60% in some samples. This agrees with previous studies showing an over 65% bioaccessibility of carotenoids [48,49].

Breads prepared by sourdough fermentation showed a lower lutein and zeaxanthin bioaccessibility compared to those fermented by conventional yeasts, suggesting that the extraction of lutein and zeaxanthin from the bread matrix occurred to a different extent depending on the fermentation procedure, or that a greater degradation of carotenoids may occur during the gastro-intestinal tract transition. Thus, it is possible that sourdough fermentation, by increasing dietary fibers through a greater solubilization of arabinoxylans [50], has influenced carotenoid removal from the bread matrix, as already reported by other authors [51].

Ferulic acid bioaccessibility was very low (less than 1% in AF breads; Table 3), in accordance with previous reports [24] and with the fact that most of it was bound to arabinoxylans and other cell wall polysaccharides, which can resist digestion in the upper gastro-intestinal tract [52]. Ferulic acid was detectable only in AF breads, while its content in SF ones was below detection limit, without significant differences between conventionally and sourdough fermented breads (Table 3). These results confirm the hypothesis that the cleavage of the ester bond in hydroxycinnamates occurs mostly in the colon, and is mediated by bacterial enzymes [52,53].

### 3.4. Anti-Inflammatory Effect of In Vitro Digested Breads

The putative anti-inflammatory effect of einkorn was evaluated comparing the soluble fraction of four digested breads, i.e. einkorn bread made using both conventional and sourdough einkorn fermentation (AFCF and AFSAF) and wheat bread made using the same types of fermentation (SFCF and SFSAF). The soluble fraction of the digested bread was supplemented to Caco-2 cells, and the ant-inflammatory effect was evaluated by measuring the secretion of IL-6 and IL-8, which are reported to be major key factors in inflammation [54]. Since bile acids have been shown to trigger oxidation [55] and to play a role in the pathogenesis of intestinal inflammation [56], in preliminary experiments cells were supplemented with the product of blank digestion, i.e. an in vitro digestion performed without food (B). In further experiments Caco-2 cells were exposed to the inflammatory stimulus (treatment with IL-1β), and then supplemented with the different digested breads or with the blank digesta. In all experiments, results obtained in supplemented cells were compared to pair matched unsupplemented (US) ones.

**Figure 5:**
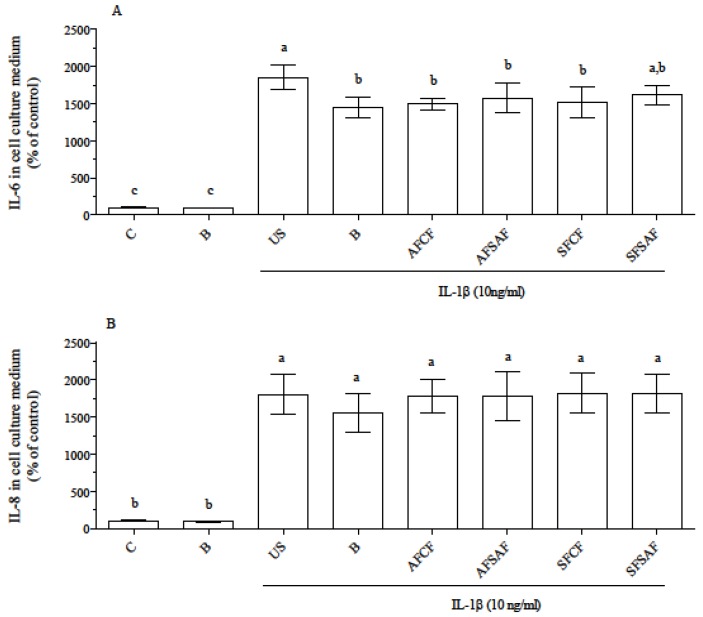
IL-6 (**A**) and IL-8 (**B**) secretion in basal condition and after IL-1β stimulation. Data are means ± SD of at least 4 samples derived from 2 independent experiments, and are expressed as the percentage of the value obtained in control, not stimulated cells (assigned as 100%). Different letters indicate statistical significance (*p* < 0.05). C = control, B = supplemented with blank digesta; US = unsupplemented; AF = ancient flour; SF = standard flour; CF = conventional fermentation; SAF = sourdough obtained from ancient flour; SSF = sourdough obtained from standard flour.

In basal condition, supplementation with the product of blank digestion did not modify IL-6 and IL-8 secretion, which was significantly increased in all cell groups upon IL-1β treatment (Figure 5) . IL-6 level increased to a higher extent in US cells than in supplemented ones except SFSAF, while the increase of IL-8 secretion was similar in all cells.

In contrast to other studies reporting a reduction of IL-8 concentration by treatment with either undigested or digested forms of polyphenol and carotenoid-rich black carrot, peel, and pomace in Caco-2 cells [57,58], we did not observe any modification in IL-8 secretion upon the inflammatory stimulus. This could be ascribable to the different concentration of supplemented bioactives. In fact, the amount of phenolic acids supplemented to cells in our study (max 30 nM, as ferulic acid equivalent) was at least 100 times lower than in [58]. High concentration of phenolic acid does not resemble a physiological, basal situation. Although they have been detected in human plasma after acute or chronic dietary intervention [59,60] and it is conceivable that they could exert an anti-inflammatory effect, the evaluation of their effectiveness was out of the scope of the present study.

**Figure 6:**
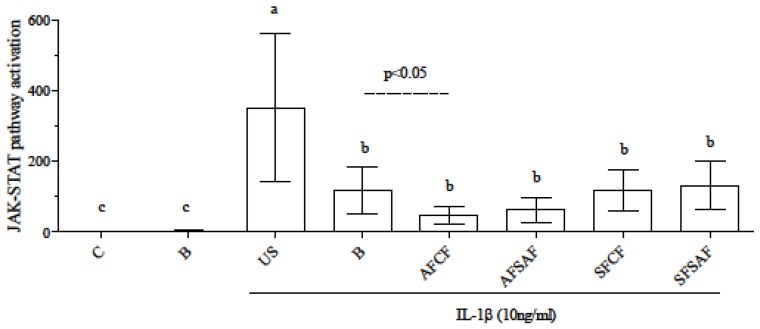
JAK-STAT pathway activation in HEK-Blue™ IL-6 cells exposed to Caco-2 cells media. Data are means ± SD of at least 4 samples derived from 2 independent experiments, and are expressed as the percentage of the value obtained in control cells (assigned as 1). Different letters indicate statistical significance (*p* < 0.05). C = control, B = supplemented with blank digesta; US = unsupplemented; AF = ancient flour; SF = standard flour; CF = conventional fermentation; SAF = sourdough obtained from ancient flour; SSF = sourdough obtained from standard flour.

IL-6 secretion upon the inflammatory stimuli was reduced by supplementation with both blank digesta and digested breads. Therefore, it was difficult to discriminate between the anti-inflammatory effect of bile acids [61] and the contribution of bread bioactives. Since IL-6 plays a key role in the induction and maintenance of gut inflammation through activation of the JAK-STAT pathway [62], to further discriminate the effect of the different bread the activation of the JAK-STAT pathway by IL-6 produced by Caco-2 cells was assayed in HEK-Blue™ IL-6 cells exposed to the medium of control and supplemented Caco-2 cells (Figure 6).

JAK-STAT activation was not detected in basal condition, while it was significantly induced by all media from IL-1β-treated Caco-2 cells. Results in HEK-Blue™ cells confirmed a lower IL-6 production in supplemented cells than US ones upon the inflammatory stimulus. Comparing inflamed, supplemented cells JAK-STAT activation appeared significantly lower in AFCF than in B ones. Therefore, although the anti-inflammatory effect seemed mainly due to the digestive fluids, bioactives in AFCF gave a contribution to the final effect.

## 4. Conclusions

The innovative development of functional bakery products requires the knowledge of the effect of formulation and process on their health-promoting compound levels. Although recent findings suggest that ancient grains may provide cardiovascular benefits [63] and reduce inflammation [64] and thus the risk of inflammation-related diseases such as irritable bowel disease [65], studies on einkorn are still relatively underdeveloped.

To the authors’ knowledge, this study represents the first integrated evaluation of the potential health benefits of einkorn-based breads compared to wheat-based ones, considering the chemical characteristics of the flours, the influence of different fermentation processes, the effect of digestion on bioactive compound bioaccessibility, and the biological effect of digested breads in cultured intestinal cells.

Our results confirm the higher carotenoid levels in einkorn than in modern wheats, which explain, at least in part, the former’s health-promoting effects. In addition, the use of sourdough fermentation seems to preserve carotenoids in the final product, despite the longer time required for processing. Significant changes in phenolic acid composition of breads also occurred, in particular a different distribution in their forms, with a clear cereal-specific effect. Moreover, the results of the in vitro digestion experiments evidenced that sourdough fermentation influences the bioaccessibility of carotenoids, with a higher retention in microbial fermented breads in comparison to yeast-fermented ones, possibly consequent to the higher solubilization of fibers mediated by lactic acid bacteria. Although the putative anti-inflammatory effect of bread in Caco-2 cells was masked by the effect of digestive fluid, experiments using HEK-Blue™ IL-6 cells evidenced a protective effect of einkorn bread made with conventional fermentation. Notably, the contribution of the gut microbiota to polyphenols transformation was not considered in this model system, and this could explain the lower effectiveness of einkorn in counteracting inflammation, already reported for other ancient grains in in vivo studies [64,65].

Notwithstanding, the results herein reported confirm einkorn as a good candidate to produce bakery products with enhanced nutritional properties.

## Figures and Tables

**Table 1:** Experimental breads.

Sample	Type of Flour	Type of Fermentation
AFCF	AF	CF
AFSAF	AF	SAF
AFSSF	AF	SSF
SFCF	SF	CF
SFSAF	SF	SAF
SFSSF	SF	SSF

AF = ancient flour; SF = standard flour; CF = conventional fermentation; SAF = sourdough obtained from ancient flour; SSF = sourdough obtained from standard flour.

**Table 2:** Ferulic and p-coumaric acid content in their FREE, SC and IB fractions in AF and SF. samples.

Flour Type	FREE	SC	IB	Total Soluble	Total
	Ferulic Acid Content (µg/g DW)				
White AF	2.2 ± 0.3a	10.0 ± 0.2a	367.8 ± 2.6a	12.2 ± 0.3a	380.0 ± 2.7a
Whole AF	1.0± 0.3b	18.0 ± 0.4b	475.8 ± 18.8b	19.0 ± 0.5b	494.8± 18.8b
SF 550 S	n.d.	4.8 ± 0.4c	126.8 ± 7.9c	4.8 ± 0.4c	131.6 ± 8.1c
SF 650 S	n.d.	3.1 ± 0.1d	117.2 ± 6.6c	3.1 ± 0.1d	120.3 ± 6.6c
SF 550 P	n.d.	4.5 ± 0.2c	124.4 ± 8.1c	4.5 ± 0.2c	128.9 ± 8.1c
SF 650 P	n.d.	5.0 ± 0.1c	131.2 ± 7.4c	5.0 ± 0.1c	136.2 ± 7.4c
	**p-Coumaric acid content (µg/g DW)**				
White AF	4.7 ± 0.9a	1.5 ± 0.3a	6.7 ± 0.9a	6.2 ± 1.0a	12.9 ± 1.3a
Whole AF	6.9 ± 0.9a	1.9 ± 0.9a	5.3 ± 0.9ab	8.8 ± 1.2a	14.1 ± 1.6a
SF 550 S	25.1 ± 1.9b	2.2 ± 0.8a	11.7 ± 1.9c	27.3 ± 2.1b	39.0 ± 2.8b
SF 650 S	26.3 ± 2.5b	2.2 ± 0.8a	8.7 ± 0.7b	28.5 ± 2.7b	37.2 ± 2.7b
SF 550 P	24.1 ± 1.3b	1.6 ± 0.6a	8.4 ± 0.9b	25.7 ± 1.4b	34.1 ± 1.7b
SF 650 P	23.9 ± 1.3b	0.3 ± 0.04a	7.7 ± 1.1b	24.2 ± 1.3b	31.9 ± 1.7b

Data represent the mean ± SD of two independent experiments with duplicate samples. Within each column, different letters indicate significant differences at *p* < 0.05. n.d. = not detected. AF = ancient flour; SF = standard flour; S = Spanish flour; P = Polish flour; SC = soluble-conjugated fraction; IB = insoluble-bound fraction.

**Table 3:** Bioaccessibility (%) of lutein, zeaxanthin and ferulic acid in digested breads.

Sample	Lutein	Zeaxanthin	Ferulic Acid
AFCF	44.1	64	0.7
AFSSF	16	13	0.6
AFSAF	8	38	0.7
SFCF	100	64	n.d.
SFSSF	52	42	n.d.
SFSAF	43	60	n.d.

Bioaccessibility for each compound was calculated as the amount detected in the soluble digested solution at the end of in vitro digestion compared to the initial content. n.d.: not detected. AF = ancient flour; SF = standard flour; CF = conventional fermentation; SAF = sourdough obtained from ancient flour; SSF = sourdough obtained from standard flour.

## References

[1] Zong G., Gao A., Hu F.B., Sun Q. (2016). Whole grain intake and mortality from all causes, cardiovascular disease, and cancer: A meta-analysis of prospective cohort studies. Circulation.

[2] Aune D., Keum N., Giovannucci E., Fadnes L.T., Boffetta P., Greenwood D.C., Tonstad S., Vatten L.J., Riboli E., Norat T. (2016). Whole grain consumption and risk of cardiovascular disease, cancer, and all cause and cause specific mortality: Systematic review and dose-response meta-analysis of prospective studies. BMJ.

[3] Ye E.Q., Chacko S.A., Chou E.L., Kugizaki M., Liu S. (2012). Greater whole-grain intake is associated with lower risk of type 2 diabetes, cardiovascular disease, and weight gain. J. Nutr..

[4] Kyrø C., Skeie G., Loft S., Landberg R., Christensen J., Lund E., Nilsson L.M., Palmqvist R., Tjønneland A., Olsen A. (2013). Intake of whole grains from different cereal and food sources and incidence of colorectal cancer in the Scandinavian HELGA cohort. Cancer Causes Control.

[5] Adom K.K., Liu R.H. (2002). Antioxidant activity of grains. J. Agric. Food Chem..

[6] Adom K.K., Sorrells M.E., Liu R.H. (2003). Phytochemical profiles and antioxidant activity of wheat varieties. J. Agric. Food Chem..

[7] Abdel-Aal E.S.M., Young J.C., Rabalski I., Hucl P., Frégeau-Reid J. (2007). Identification and quantification of seed carotenoids in selected wheat species. J. Agric. Food Chem..

[8] Perez-Jimenez J., Neveu V., Vos F., Scalbert A. (2010). Systematic analysis of the content of 502 polyphenols in 452 foods and beverages: An application of the Phenol−Explorer database. J. Agric. Food Chem..

[9] Abdel-Aal E.S.M., Young J.C., Wood P.J., Rabalski I., Hucl P., Fregeau-Reid J. (2002). Einkorn: A potential candidate for developing high lutein wheat. Cereal Chem..

[10] Humphries J.M., Khachik F. (2003). Distribution of lutein, zeaxanthin and related geometrical isomers in fruit, vegetables, wheat and pasta products. J. Agric. Food Chem..

[11] Sosulski F., Krygier K., Hogge L. (1982). Free, esterified, and insoluble-bound phenolic acids. 3. Composition of phenolic acids in cereal and potato flour. J. Agric. Food Chem..

[12] Poutanen K., Shepherd R., Shewry P.R., Delcour J.A., Bjorck I., Van Der Kamp J.W. (2008). Beyond whole grain: The European HEALTH GRAIN project aims at healthier cereal foods. Cereal Foods World.

[13] Slavin J. (2003). Why whole grains are protective: Biological mechanisms. Proc. Nutr. Soc..

[14] Bordoni A., Danesi F., Di Nunzio M., Taccari A., Valli V. (2016). Ancient wheat and health: A legend or the reality? A review on KAMUT khorasan wheat. Int. J. Food Sci. Nutr..

[15] Hidalgo A., Brandolini A. (2014). Nutritional properties of einkorn wheat (*Triticum monococcum* L.). J. Sci. Food Agric..

[16] Molberg Ø., Uhlen A.K., Jensen T., Flæte N.S., Fleckenstein B., Arentz-Hansen H., Raki M., Lundin K.E.A., Sollid L.M. (2005). Mapping of gluten T-cell epitopes in the bread wheat ancestors: Implication for celiac disease. Gastroenterology.

[17] Sánchez-Pardo M.E., Blancas-Nápoles J.A., Vázquez-Landaverde P.A., Nari A., Taglieri I., Ortiz-Moreno A., Mayorga-Reyes L., Sanmartin C., Bermúdez-Humarán L.G., Torres-Maravilla E. (2016). The use of Mexican xaxtle as leavening agent in Italian straight dough bread making to produce pulque bread. Agrochimica.

[18] Venturi F., Sanmartin C., Taglieri I., Nari A., Andrich G., Zinnai A. (2016). Effect of the baking process on artisanal sourdough bread-making: A technological and sensory evaluation. Agrochimica.

[19] Ganzle M.G. (2014). Enzymatic and bacterial conversions during sourdough fermentation. Food Microbiol..

[20] Gobbetti M., Rizzello G.C., Di Cagno R., De Angelis M. (2014). How the sourdough may affect the functional features of leavened baked goods. Food Microbiol..

[21] Venturi F., Sanmartin C., Taglieri I., Nari A., Andrich G., Terzuoli E., Donnini S., Nicolella C., Zinnai A. (2017). Development of phenol-enriched olive oil with phenolic compounds extracted from wastewater produced by physical refining. Nutrients.

[22] Turpin W., Renaud C., Avallone S., Hammoumi A., Guyot J.-P., Humblot C. (2016). PCR of crtNM combined with analytical biochemistry: An efficient way to identify carotenoid producing lactic acid bacteria. Syst. Appl. Microbiol..

[23] Reboul E., Richelle M., Perrot E., Smoulins-Malezet C., Pirisi V., Borel P. (2006). Bioaccessibility of carotenoids and vitamin E from their main dietary sources. J. Agric. Food Chem..

[24] Anson N.M., van den Berg R., Havenaar R., Bast A., Haenen G.R.M.M. (2009). Bioavailability of ferulic acid is determined by its bioaccessibility. J. Cereal Sci..

[25] Viadel Crespo B., Rivera Patiño J.D., Navarro Fayos M.T., Tenllado Llavador I., Carreres Malonda J.E., García Reverter J., Blasco Piquer M., Subirats Huerta S. (2012). Equipo modular de digestión in vitro. Patent.

[26] Marteau P., Flourié B., Pochart P., Chastang C., Desjeux J.F., Rambaud J.C. (1990). Role of the microbial lactose EC 3.2.123, activity from yoghurt on the intestinal absorption of lactose: An in vivo study in lactose-deficient humans. Br. J. Nutr..

[27] Minekus M., Marteau P., Havenaar R., Huis Veld J.H.J. (1995). A multicompartmental dynamic computer-controlled model simulating the stomach and the small intestine. ATLA.

[28] Bengtsson A., Larsson M., Svanberg U. (2009). In vitro bioaccessibility of β-carotene from heat-processed orange-fleshed sweet potato. J. Agric. Food Chem..

[29] Moore J., Hao Z., Zhou K., Luther M., Costa J., Yu L. (2005). Carotenoid, tocopherol, phenolic acid, and antioxidant properties of Maryland-grown soft wheat. J. Agric. Food Chem..

[30] Mattila P., Pihlava J.M., Hellström J. (2005). Contents of phenolic acids, alkyl- and alkenylresorcinols, and avenanthramides in commercial grain products. J. Agric. Food Sci..

[31] Hidalgo A., Brandolini A., Pompei C., Piscozzi R. (2006). Carotenoids and tocols of einkorn wheat (*Triticum monococcum* ssp. *monococcum* L.). J. Cereal Sci..

[32] Brandolini A., Castoldi P., Plizzari L., Hidalgo A. (2013). Phenolic acid composition, total polyphenols content and antioxidant activity of *Triticum monococcum*, *Triticum turgidum* and *Triticum aestivum*: A two-years evaluation. J. Cereal Sci..

[33] Abdel-Aal E.S.M., Rabalski I. (2008). Bioactive compounds and their antioxidant capacity in selected primitive and modern wheat species. Open Agric. J..

[34] Hidalgo A., Brandolini A. (2008). Protein, ash, lutein and tocols distribution in einkorn (*Triticum monococcum* L. subsp. *Monococcum*) seed fractions. Food Chem..

[35] Adom K.K., Sorrells M.E., Liu R.H. (2005). Phytochemicals and antioxidant activity of milled fractions of different wheat varieties. J. Agric. Food Chem..

[36] Leenhardt F., Lyan B., Rock E., Boussard A., Potus J., Chanliaud E., Remesy C. (2006). Wheat lipoxygenase activity induces greater loss of carotenoids than Vitamin E during breadmaking. J. Agric. Food Chem..

[37] Leenhardt F., Lyan B., Rock E., Boussard A., Potus J., Chanliaud E., Remesy C. (2006). Genetic variability of carotenoid concentration, and lipoxygenase and peroxidase activities among cultivated wheat species and bread wheat varieties. Eur. J. Agron..

[38] Cevoli C., Gianotti A., Troncoso R., Fabbri A. (2015). Quality evaluation by physical tests of a traditional Italian flat bread Piadina during storage and shelf-life improvement with sourdough and enzymes. Eur. Food Res. Technol..

[39] Ferri M., Serrazanetti D.I., Tassoni A., Baldissarri M., Gianotti A. (2016). Improving functional and technological profile of cereal fermented foods by *Lactobacillus plantarum* strains selected via a metabolomics approach. Food Res. Int..

[40] Garrido-Fernandez J., Maldonado-Barragan A., Caballero-Guerrero B., Hornero-Mendez D., Ruiz-Barba J.L. (2010). Carotenoid production in *Lactobacillus plantarum*. Int. J. Food Microbiol..

[41] Breithaupt D.E., Schwack W., Wolf G., Hammes W.P. (2001). Characterization of the triterpenoid diaponeurosporene and its isomers in food-associated bacteria. Eur. Food Res. Technol..

[42] Katina K., Laitila A., Jovonen R., Liukkonen K.-H., Kariluoto S., Piironen V., Landgerg R., Åman P., Poutanen K. (2007). Bran fermentation as a means to enhance technological properties and bioactivity of rye. Food Microbiol..

[43] Boskov Hansen H., Andreasen M.G., Nielsen M.M., Larsen L.M., Bach Knudsen K.E., Meyer A.S., Christensen L.P., Hansen Å. (2002). Changes in dietary fibre, phenolic acids and activity of endogenous enzymes during rye bread-making. Eur. Food Res. Technol..

[44] Hole A.S., Rud I., Grimmer S., Sigl S., Narvhus J., Sahlstrøm S. (2012). Improved bioavailability of dietary phenolics in whole grain barley and oat groat following fermentation with probiotic *Lactobacillus acidophilus*, *Lactobacillus johnsonii*, and *Lactobacillus reuteri*. J. Agric. Food Chem..

[45] Svensson L., Sekwati-Monang B., Lutz D.L., Schieber A., Ganzle M.G. (2010). Phenolic acids and flavonoids in nonfermented and fermented red sorghum *Sorghum bicolor* L., Moench. J. Agric. Food Chem..

[46] Gobbetti M., De Angelis M., Corsetti A., Di Cagno R. (2005). Biochemistry and physiology of sourdough lactic acid bacteria. Trends Food Sci. Technol..

[47] Makris D.P., Boskou G., Andrikopoulos N.K. (2007). Polyphenolic content and in vitro antioxidant characteristics of wine industry and other agri-food solid waste extracts. J. Food Compos. Anal..

[48] Read A., Wright A., Abdel-Aal E.S.M. (2015). In vitro bioaccessibility and monolayer uptake of lutein from wholegrain baked foods. Food Chem..

[49] Courraud J., Berger J., Cristol J.P., Avallone S. (2013). Stability and bioaccessibility of different forms of carotenoids and vitamin A during in vitro digestion. Food Chem..

[50] Katina K., Juvonen R., Laitila A., Flander L., Nordlund E., Kariluoto S., Piironen V., Poutanen K. (2012). Fermented wheat bran as a functional ingredient in baking. Cereal Chem..

[51] O’Connell O., Ryan L., O’Sullivan L., Aherne-Bruce S.A., O’Brien N.M. (2008). Carotenoid micellarization varies greatly between individual and mixed vegetables with or without the addition of fat or fiber. Int. J. Vitam. Nutr. Res..

[52] Zhao Z., Egashira Y., Sanada H. (2003). Digestion and absorption of ferulic acid sugar esters in rat gastrointestinal tract. J. Agric. Food Chem..

[53] Kroon P.A., Faulds C.B., Ryden P., Robertson J.A., Williamson G. (1997). Release of covalently bound ferulic acid from fiber in the human colon. J. Agric. Food Chem..

[54] Laveti D., Kumar M., Hemalatha R., Sistla R., Naidu V.G., Talla V., Verma V., Kaur N., Nagpal R. (2013). Anti-inflammatory treatments for chronic diseases: A review. Inflamm. All. Drug Targets.

[55] Araki Y., Katoh T., Ogawa A., Bamba S., Andoh A., Koyama S., Fujiyama Y., Bamba T. (2005). Bile acid modulates transepithelial permeability via the generation of reactive oxygen species in the Caco-2 cell line. Free Radic. Biol. Med..

[56] Di Toro R., Campana G., Murari G., Spampinato S. (2000). Effects of specific bile acids on c-fos messenger RNA levels in human colon carcinoma Caco-2 cells. Eur. J. Pharm. Sci..

[57] Kaulmann A., Bohn T. (2014). Carotenoids, inflammation, and oxidative stress—Implications of cellular signaling pathways and relation to chronic disease prevention. Nutr. Res..

[58] Kamiloglu S., Grootaert C., Capanoglu E., Ozkan C., Smagghe G., Raes K., Van Camp J. (2017). Anti-inflammatory potential of black carrot (*Daucus carota* L.) polyphenols in a co-culture model of intestinal Caco-2 and endothelial EA.hy926 cells. Mol. Nutr. Food Res..

[59] Marmet C., Actis-Goretta L., Renouf M., Giuffrida F. (2014). Quantification of phenolic acids and their methylates, glucuronides, sulfates and lactones metabolites in human plasma by LC-MS/MS after oral ingestion of soluble coffee. J. Pharm. Biomed. Anal..

[60] McKay D.L., Chen C.Y., Zampariello C.A., Blumberg J.B. (2015). Flavonoids and phenolic acids from cranberry juice are bioavailable and bioactive in healthy older adults. Food Chem..

[61] Ward J.B.J., Lajczak N.K., Kelly O.B., O’Dwyer A.M., Giddam A.K., Ní Gabhann J., Franco P., Tambuwala M.M., Jefferies C.A., Keely S. (2017). Ursodeoxycholic acid and lithocholic acid exert anti-inflammatory actions in the colon. Am. J. Physiol. Gastrointest. Liver Physiol..

[62] Fantini M.C., Pallone F. (2008). Cytokines: From gut inflammation to colorectal cancer. Curr. Drug Targets..

[63] Sereni A., Cesari F., Gori A.M., Maggini N., Marcucci R., Casini A., Sofi F. (2017). Cardiovascular benefits from ancient grain bread consumption: Findings from a double-blinded randomized crossover intervention trial. Int. J. Food Sci. Nutr..

[64] Benedetti S., Primiterra M., Tagliamonte M.C., Carnevali A., Gianotti A., Bordoni A., Canestrari F. (2012). Counteraction of oxidative damage in the rat liver by an ancient grain (Kamut brand khorasan wheat). Nutrition.

[65] Sofi F., Whittaker A., Gori A.M., Cesari F., Surrenti E., Abbate R., Gensini G.F., Benedettelli S., Casini A. (2014). Effect of Triticum turgidum subsp. turanicum wheat on irritable bowel syndrome: A double-blinded randomised dietary intervention trial. Br. J. Nutr..

